# A randomized control study on anesthetic effects of flurbiprofen axetil combined with propofol on patients with liver cancer receiving microwave ablation

**DOI:** 10.12669/pjms.36.6.2091

**Published:** 2020

**Authors:** Xingshi Gu, Qiang Yuan, Jian Zhang, Yawen Yang

**Affiliations:** 1 Xingshi Gu, Department of Interventional Radiology, Second Affiliated Hospital of Soochow University, Suzhou 215000, Jiangsu Province, P. R. China; 2 Qiang Yuan, Department of Interventional Radiology, Second Affiliated Hospital of Soochow University, Suzhou 215000, Jiangsu Province, P. R. China; 3 Jian Zhang, Department of Interventional Radiology, Second Affiliated Hospital of Soochow University, Suzhou 215000, Jiangsu Province, P. R. China; 4 Yawen Yang, Department of Anesthesiology, First Affiliated Hospital of Soochow University, Suzhou 215000, Jiangsu Province, P. R. China

**Keywords:** Anesthesia, Flurbiprofen axetil, Liver cancer, Microwave ablation, Propofol

## Abstract

**Objective:**

To evaluate the anesthetic effects of flurbiprofen axetil combined with propofol on patients with liver cancer receiving microwave ablation.

**Methods:**

Sixty patients (SA grade: I-II) who underwent microwave ablation for liver cancer in our hospital from May 2018 to May 2019 were selected and randomly divided into a study group and a control group (n=30) that were anesthetized through target-controlled infusion of propofol combined with intravenous infusion of flurbiprofen axetil and target-controlled infusion of propofol alone, respectively. The mean arterial pressure (MAP), heart rate (HR), blood oxygen saturation (SpO_2_), numerical rating scale (NRS) score, adverse reactions and anesthetic effects (induction time, recovery time) of the two groups were compared before anesthesia (T0), at the beginning of puncture (T1), at the beginning of microwave ablation (T2), at the end of microwave ablation (T3) and one hour after surgery (T4).

**Results:**

MAP and HR of the study group were higher than those of the control group (P<0.05) at T2. There was no difference in SpO_2_ between the two groups (P>0.05). The anesthesia induction time and recovery time of the study group were significantly shorter than those of the control group (P<0.05). There was no difference in the NRS score between the two groups at T1 (P>0.05), but the study group had lower scores at T2-T4 (P<0.05). The incidence rate of postoperative adverse reactions was 13.33% in the study group and 46.67% in the control group, with a significant difference (P<0.05).

**Conclusion:**

Flurbiprofen axetil in combination with propofol exert evident anesthetic effects on patients with liver cancer receiving microwave ablation. The time of preoperative anesthesia induction and postoperative recovery time can be markedly shortened, and intraoperative vital signs can be maintained stable. This method is thus worthy of clinical promotion.

## INTRODUCTION

Percutaneous microwave ablation is generally used to treat liver cancer, during which a microwave probe is employed to puncture the vicinity of liver cancer tissue and to promote protein coagulation using highly focused heat in targeting areas, finally causing irreversible damage to cancer cells and deactivation.^1^ However, due to local high-temperature stimulation, patients tend to move during surgery under local anesthesia, which results in displacement of the microwave probe and unnecessary danger. Therefore, intravenous anesthesia is commonly utilized.^2^ Propofol is commonly used for anesthesia induction and maintenance in clinical practice, but it has unstable anesthetic effects.^3^ As a non-steroidal targeting analgesic agent, flurbiprofen axetil is generally used after surgery, but its combinations with other drugs before surgery have seldom been reported.^4^ This study was to assess the anesthetic effects of flurbiprofen axetil combined with propofol on patients with liver cancer receiving microwave ablation.

## METHODS

### Baseline clinical data

The sample size was calculated according to the two independent-sample method:


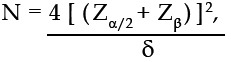


where N is the sample size, Z_α/2_ is the Z value (which is 1.96) corresponding to α of 0.05, Z_β_ is the Z value (which is 0.84) corresponding to type II error probability β of 0.20, and δ is the allowable error (which is generally 8.4). The sample size N was thus calculated as 25. In other words, over 25 cases should be included for each group. Therefore, we herein included 30 cases for each group.

Sixty patients who underwent microwave ablation for liver cancer in our hospital from May 2018 to May 2019 were selected. Inclusion criteria: ASA grade I-II; Child-Pugh grade A and B.^5^ Exclusion criteria: With severe heart, lung or renal dysfunction; with atrioventricular block; with abnormal thyroid function; with long-term use of analgesics or sedatives; with allergy to anesthetics. This study has been approved by the ethics committee of our hospital at 4^th^ May, 2018 (registration number: CH201801280124), and written informed consent has been obtained from all patients. The patients were divided into a study group and a control group (n=30) by the random number table method. The study group consisted of 14 males and 16 females aged 35-70 years old, (48.72 ± 5.71) on average. The mean BMI was (65.71 ± 4.58) kg/m^2^. The control group comprised 15 males and 15 females aged 36-70 years old, (49.55 ± 4.81) on average. The mean BMI was (66.44 ± 3.78) kg/m^2^. The gender ratio, age and BMI of the two groups were not statistically significant (P>0.05).

### Methods

After entering the operating room, the two groups were routinely monitored for mean arterial pressure (MAP), heart rate (HR) and blood oxygen saturation (SpO_2_). The venous access was opened, and maintenance infusion was performed with sodium chloride solution (5 ml/min).

The control group was given target-controlled infusion of propofol by using an infusion pump. The plasma concentration was set to 3 μg/ml. The intraoperative dose was adjusted in the range of 0.3-0.5 μg/ml. BIS was maintained at 40~50. The procedure was stopped five minutes before the end of surgery.

The study group was anesthetized by target-controlled infusion of propofol in combination with flurbiprofen axetil, i.e. intravenous infusion of flurbiprofen axetil 15 minutes before target-controlled infusion of propofol at the dose of 2.5 mg/kg. The remaining procedure was the same as that of the control group.

If the two groups suffered from intraoperative respiratory depression, oxygen uptake was conducted using a mask. If necessary, artificial mechanical ventilation was carried out. In the case of severe hypotension (ΔMAP >30%), ephedrine was intravenously infused at the dose of six mg/time. Atropine was injected (0.3 mg each time) when HR was <50 bpm.

### Observation indices

MAP, HR, SpO_2_, numerical rating scale (NRS) score, adverse reactions and anesthetic effects (induction time, recovery time) of the two groups were compared before anesthesia (T0), at the beginning of puncture (T1), at the beginning of microwave ablation (T2), at the end of microwave ablation (T3) and 1 h after surgery (T4).

### NRS

NRS was used for evaluation: 0 point for no pain; 1~3 points for mild pain; 4~6 points for tolerable pain affecting sleep; 7~10 points for intolerable pain.^6^

### Statistical analysis

All data were statistically analyzed by SPSS21.0 software. The categorical data were compared by the t test and expressed as (`x ± s). The numerical data were subjected to the Chi-square test. P<0.05 was considered statistically significant.

## RESULTS

### Vital sign changes

MAP and HR of both groups decreased at T1 and then increased, but the SpO_2_ values of the two groups hardly changed throughout anesthesia. MAP and HR of the study group were higher than those of the control group (P<0.05) at T2. There was no difference in SpO_2_ between the two groups (P>0.05) (Table-I).

**Table-I:** Vital sign changes (x̄±s)

Index	Study group (n=30)	Control group (n=30)	t value	P value
***MAP (mmHg)***				
T0	97.14±5.14	97.81±6.88	0.43	0.67
T1	89.11±6.82	88.19±5.55	0.57	0.57
T2	101.25±7.26	93.14±9.58	3.70	0.00
T3	105.16±5.47	102.91±5.88	1.53	0.13
T4	103.57±7.76	104.16±4.19	0.37	0.72
***HR (bpm)***				
T0	76.14±5.74	77.58±6.88	0.88	0.38
T1	73.25±6.25	74.19±5.58	0.61	0.54
T2	80.14±6.81	67.81±6.74	7.05	0.00
T3	92.16±7.71	91.14±5.91	0.58	0.57
T4	77.64±9.54	80.68±6.28	1.46	0.15
***SpO_2_ (%)***				
T0	98.14±5.88	98.81±4.91	0.48	0.63
T1	99.58±7.91	99.87±5.94	0.16	0.87
T2	99.84±6.88	99.71±7.58	0.07	0.94
T3	99.71±6.17	99.54±8.81	0.09	0.93
T4	99.88±6.16	99.25±9.14	0.31	0.76

### Anesthetic effects

The anesthesia induction time and recovery time of the study group were significantly shorter than those of the control group (P<0.05). The NRS scores of both groups first increased and then dropped. There was no difference in the NRS score between the two groups at T1 (P>0.05), but the study group had lower scores at T2-T4 (P<0.05) (Table-II).

**Table-II:** Anesthetic effects (x̄±s)

Group	Induction time (s)	Recovery time (min)	NRS score (point)			

			T1	T2	T3	T4
Control (n=30)	74.14±5.71	8.17±2.85	0.54±0.06	4.01±1.21	3.94±1.01	2.24±1.06
Study (n=30)	62.14±4.11	5.16±2.14	0.55±0.04	2.14±1.05	2.06±0.96	0.67±0.16
t value	9.34	4.63	0.76	6.39	7.39	8.02
P value	0.00	0.00	0.45	0.00	0.00	0.00

### Postoperative adverse reactions

The incidence rate of postoperative adverse reactions including bradycardia, delirium, hypotension, nausea, vomiting, headache and respiratory depression was 13.33% in the study group and 46.67% in the control group, with a significant difference (P<0.05) (Table-III).

**Table-III:** Postoperative adverse reactions (x̄±s)

Group	Bradycardia	Delirium	Hypotension	Nausea and vomiting	Headache	Respiratory depression	Incidence rate	χ^2^ value	P value
Control (n=30)	2 (6.67)	2 (6.67)	2 (6.67)	1 (3.33)	1 (3.33)	6 (20.00)	14 (46.67)	7.94	0.00
Study (n=30)	1 (3.33)	0	1 (3.33)	1 (3.33)	0	1 (3.33)	4 (13.33)
Z value	5.47						-	-	-
P value	0.00						-	-	-

## DISCUSSION

For liver cancer patients undergoing percutaneous microwave ablation, propofol is usually injected intravenously for anesthesia. Propofol is a short-acting intravenous anesthetic. After the medication, the patient enters the anesthesia state for a short time, but the time to maintain the anesthetic effect is relatively short.^7^ Generally, targeted injection is used to perform propofol anesthesia, and dose supplementation is performed in small quantities for several times.^8^ However, even with this method, the amount of propofol injected by patients is still relatively large. A large amount of propofol can cause hemodynamic fluctuations in patients, leading to adverse reactions such as respiratory depression and respiratory function suspension.^9^ Besides, propofol has a direct dilating effect on surrounding blood vessels, which can decrease the blood pressure and heart rate and even induce atrioventricular block in extreme cases. Meanwhile, the analgesic effect of propofol is weak.^10^ Therefore, propofol is often combined with other anesthetic and analgesic drugs in clinical practice.^11^

Flurbiprofen axetil, as a prodrug of flurbiprofen, has mainly been used for cancer and postoperative analgesia.^12^ This drug is carried by lipid microspheres, targeted to tumor or trauma sites and released to generate flurbiprofen through rapid hydrolysis by carboxylesterase. Afterwards, flurbiprofen allows analgesia by inhibiting prostaglandin synthesis.^13^ In this study, both T2 MAP and HR of the study group were higher than those of the control group (P<0.05), but there was no significant difference in MAP and HR between the two groups at other time periods (P>0.05). The results showed that the anesthetic drugs used in the two groups had little effect on the vital signs of the patients, but when the patients underwent microwave ablation, they were still affected, and the vital signs changed, while the remaining time was short and the vital signs of the patients in the two groups did not change. Possibly, propofol was used consistently in the two groups of patients, with a recognized safety. The safe dosage of propofol is 3 ml/kg, which has the most significant and safest analgesic effect for patients.^14^ The advantage of flurbiprofen axetil in combination is that it has no central inhibitory effect or significant respiratory inhibitory effect on patients.^15^ After the treatment, patients’ breathing is not affected, so indices related to respiratory function such as heart rate, arterial pressure, and oxygen saturation are not affected.

The reason why the patients in the study group were injected with flurbiprofen 15 minutes before surgery was that flurbiprofen began to take effect 10 min after entering the body. The patients in the study group were anesthetized with propofol and flurbiprofen in a target-controlled infusion, whose induction time and recovery time were significantly shorter than those in the control group (P<0.05), indicating that the combination of the two drugs had a significant effect on anesthesia and analgesia. The NRS scores showed that the NRS scores of the study group patients in the T2-T4 stage were lower than those of the control group (P<0.05), suggesting that the analgesic effect of the study group was more significant. Flurbiprofen axetil, a precursor non-steroidal targeted analgesic drug, is generally used for postoperative analgesia. In pharmacology, flurbiprofen axetil uses lipid microspheres as a carrier, which contains propionic acid non-corporeal anti-inflammatory drugs with carboxyl structure. Lipid microspheres have a similar distribution to liposomes, and will be selectively attached to the trauma site after entering the human body. Lipid microspheres release flurbiprofen axetil, which is generated into flurbiprofen under the action of carboxyl esterase, and acts on the cut.^16^ Its mechanism of action is to inhibit the production of cyclooxygenase, reduce the content of prostaglandins and the traumatic sensation and pain perception of peripheral nerves through the action of the central nervous system, producing analgesic effects. Meanwhile, since there is no central inhibitory effect, it will not affect the recovery time of patients.^17^

This study also compared the adverse reactions of patients after anesthesia. In this study, the incidence of postoperative adverse reactions was 13.33% in the study group and 46.67% in the control group at identical BIS, between which the former was significantly lower than the latter (P<0.05). Therefore, the anesthesia method adopted by the study group was safe and reliable. Through analysis, the reasons may be related to the following factors. Flurbiprofen axetil used in this study had less effect on respiratory depression and heart rate of patients, fewer patients were evoked with spontaneous breathing and fewer times during the operation, and the depth of intraoperative anesthesia and sedation was stable. Probably, flurbiprofen axetil was used before the propofol anesthesia. When the patient was given propofol anesthesia again after the onset of effect, the patient received the drug faster because of the sedative effect of flurbiprofen axetil, so that the vital signs of the patient’s body and the depth of anesthesia were maintained in a relatively stable state.^18^ In contrast, the control group was anesthetized with single propofol. Due to the short duration of drug anesthesia depth, although target-controlled infusion technology was adopted, the patients would inevitably experience instability in the depth of anesthesia, which led to body movements when respiratory inhibitors were called, affecting the surgery. Anesthetic drugs are mostly used for analgesia through central nervous system depression, opioid receptor agonism, α2 adrenergic receptor agonism, etc. Therefore, delirium, nausea, vomiting, and headache are all adverse reactions produced by traditional narcotics.^19^ Herein, there were fewer adverse reactions after surgery in the study group, mainly because flurbiprofen axetil had no central effect and a strong sedative effect, which could significantly reduce the adverse reactions caused by propofol and after anesthesia.

### Limitations of the study

This is a single-center study with small sample size. In the future, we will perform multicenter studies with larger sample sizes to assess the anesthetic effects of flurbiprofen axetil combined with propofol on patients with liver cancer receiving microwave ablation.

## CONCLUSION

In summary, the use of flurbiprofen axetil combined with propofol for microwave ablation in patients with liver cancer has a significant anesthetic effect. The preoperative anesthesia induction time and postoperative recovery time are effectively shortened, the intraoperative vital signs are stable, and the pain control effect is good, which can be used for clinical promotion.

### Authors’ Contributions:

**XG & YY:** Study design and significant manuscript revision.

**XG, QY & JZ:** Manuscript drafting, clinical data collection and analysis.

**XG, QY, JZ & YY:** Approval of manuscript submission.

**YY** is responsible and accountable for the accuracy or integrity of the study.
